# Longer-term effectiveness of a heterologous coronavirus disease 2019 (COVID-19) vaccine booster in healthcare workers in Brazil

**DOI:** 10.1017/ash.2023.173

**Published:** 2023-06-22

**Authors:** Alexandre R. Marra, João Luiz Miraglia, Daniel Tavares Malheiro, Yang Guozhang, Vanessa Damazio Teich, Elivane da Silva Victor, João Renato Rebello Pinho, Adriana Cypriano, Laura Wanderly Vieira, Miria Polonio, Rafael Herrera Ornelas, Solange Miranda de Oliveira, Flavio Araujo Borges, Silvia Cristina Cassiano Oler, Victória Catharina Volpe Ricardo, Aline Miho Maezato, Gustavo Yano Callado, Guilherme de Paula Pinto Schettino, Ketti Gleyzer de Oliveira, Rúbia Anita Ferraz Santana, Fernanda de Mello Malta, Deyvid Amgarten, Ana Laura Boechat, Takaaki Kobayashi, Jorge L. Salinas, Michael B. Edmond, Luiz Vicente Rizzo

**Affiliations:** 1 Hospital Israelita Albert Einstein, São Paulo, Brazil; 2 Department of Internal Medicine, University of Iowa, Iowa City, Iowa, United States; 3 Stanford University, Stanford, California, United States; 4 West Virginia University School of Medicine, Morgantown, West Virginia, United States

## Abstract

**Objective:**

To compare the long-term vaccine effectiveness between those receiving viral vector [Oxford-AstraZeneca (ChAdOx1)] or inactivated viral (CoronaVac) primary series (2 doses) and those who received an mRNA booster (Pfizer/BioNTech) (the third dose) among healthcare workers (HCWs).

**Methods:**

We conducted a retrospective cohort study among HCWs (aged ≥18 years) in Brazil from January 2021 to July 2022. To assess the variation in the effectiveness of booster dose over time, we estimated the effectiveness rate by taking the log risk ratio as a function of time.

**Results:**

Of 14,532 HCWs, coronavirus disease 2019 (COVID-19) was confirmed in 56.3% of HCWs receiving 2 doses of CoronaVac vaccine versus 23.2% of HCWs receiving 2 doses of CoronaVac vaccine with mRNA booster (*P* < .001), and 37.1% of HCWs receiving 2 doses of ChAdOx1 vaccine versus 22.7% among HCWs receiving 2 doses of ChAdOx1 vaccine with mRNA booster (*P* < .001). The highest vaccine effectiveness with mRNA booster was observed 30 days after vaccination: 91% for the CoronaVac vaccine group and 97% for the ChAdOx1 vaccine group. Vacine effectiveness declined to 55% and 67%, respectively, at 180 days. Of 430 samples screened for mutations, 49.5% were SARS-CoV-2 delta variants and 34.2% were SARS-CoV-2 omicron variants.

**Conclusions:**

Heterologous COVID-19 vaccines were effective for up to 180 days in preventing COVID-19 in the SARS-CoV-2 delta and omicron variant eras, which suggests the need for a second booster.

In 2023, even with the considerable distribution and access to coronavirus disease 2019 (COVID-19) vaccines, individuals are still at risk of infection.^1,2^ It has been proven that vaccination can reduce the incidence of serious outcomes, such as hospitalization and death.^3,4^

Healthcare workers have frequent exposure to COVID-19.^5,6^ Even though the availability of personal protective equipment (PPE) is helpful in the prevention of infection,^7^ access may be limited in resource-poor settings.

COVID-19 in this new phase of the pandemic can be partly explained by the relaxation of prevention measures that many countries had adopted in 2020 and 2021.^8–10^ Some studies have concluded that a booster vaccine dose raises vaccine effectiveness considerably.^11,12^ In Brazil, a significant share of the population received 2 doses of Oxford-AstraZeneca [ChAdOx1] or CoronaVac instead of mRNA vaccines because these vaccines were available first in many countries. In October 2021, our institution started administering mRNA vaccine boosters (Pfizer/BioNTech) for HCWs who had received either ChAdOx1 or CoronaVac as their primary series of 2 doses.^10^

In our previous study, we assessed the short-term (≤3 months) vaccine effectiveness of an mRNA vaccine booster following 2 doses of ChAdOx1 or CoronaVac against laboratory-confirmed COVID-19 among HCWs in Brazil.^13^ In this study, we have continued our evaluation of the effectiveness of an mRNA booster; we have extended the analysis over an 18-month period to evaluate the longer-term vaccine effectiveness in the SARS-CoV-2 delta and omicron variant eras.

## Methods

### Population and setting

We conducted a retrospective cohort study of all adult HCWs (aged ≥18 years) working at the Hospital Israelita Albert Einstein (HIAE) between January 1, 2021, and July 30, 2022. The HIAE is a Brazilian nonprofit healthcare, educational, and research organization headquartered in São Paulo. It manages diverse services from primary to tertiary care, in the public and private healthcare sectors, and it operates 40 healthcare units, mainly in the state of São Paulo. In 2020, the HIAE had 700,000 emergency department visits, 900,000 outpatient visits, and 70,000 hospital discharges. Since the beginning of the COVID-19 pandemic, HCWs with COVID-19 symptoms had access to free-of-charge SARS-CoV-2 RT-PCR testing conducted by the institution’s laboratory.

We included HCWs who completed at least 2 doses of either ChAdOx1 or CoronaVac vaccines, and we compared vaccine effectiveness in those who received an optional booster dose of mRNA vaccine to those who did not. HCWs were followed for 18 months (10 months following the booster dose). We excluded HCWs who no longer worked at HIAE, received just 1 dose of any COVID-19 vaccine, and received other combinations of COVID-19 vaccines (eg, Janssen vaccine + Pfizer/BioNTech vaccine), or received 4 doses of a COVID-19 vaccine (Supplementary Appendix 1).

### Real-time polymerase chain reaction (RT-PCR) methodologies for SARS-CoV-2 detection

Diagnostic confirmation for COVID-19 was performed using RT-PCR on specimens obtained via nasopharyngeal swab, according to the protocol instituted at HIAE. The following RT-PCR kits were utilized: XGEN MASTER COVID-19 (Mobius, Pinhais, Paraná, Brazil), cobas SARS-CoV-2 Test (Roche Molecular Systems, Branchburg, NJ), Xpert Xpress SARS-CoV-2 (Cepheid, Sunnyvale, CA, USA), and Abbott RealTime SARS-CoV-2 (Abbott Molecular, Des Plaines, IL).

### Next-generation sequencing of the viral full-length genome

We extracted total nucleic acid from naso-oropharyngeal (NOP) swab samples with the QIAamp Viral RNA Mini kit (QIAGEN, Hilden, Germany). After purification and concentration, DNAse I treatment, and depletion of human ribosomal RNA, samples were submitted to random amplification.^14^ Preparation of sequencing libraries for the Illumina platform was carried out with DNA Prep (Illumina, San Diego, CA) using the random 2-step PCR amplification product as input. Libraries were quantified using the Qubit instrument (Thermo Fisher Scientific, Waltham, MA) and were loaded on the NextSeq 550 equipment (Illumina) for sequencing with MID 300 paired-end reads (Illumina).

### Outcome measures and statistical analyses

Laboratory-confirmed COVID-19 was considered the primary outcome for calculating vaccine effectiveness. RT-PCR testing for the diagnosis of COVID-19 was performed only on symptomatic HCWs. Hospitalization related to COVID-19, length of stay, ICU admission, mechanical ventilation, and death were considered secondary outcomes. The vaccination status of all study participants and SARS-CoV-2 RT-PCR results of symptomatic HCWs were obtained from institutional electronic records. For those vaccinated, the initial follow-up date was 14 days after the second or the third vaccine dose. The last date was defined as the date COVID-19 was diagnosed, or up to July 30, 2022, for the censored cases without a positive diagnosis of COVID-19.

**Figure 1.**
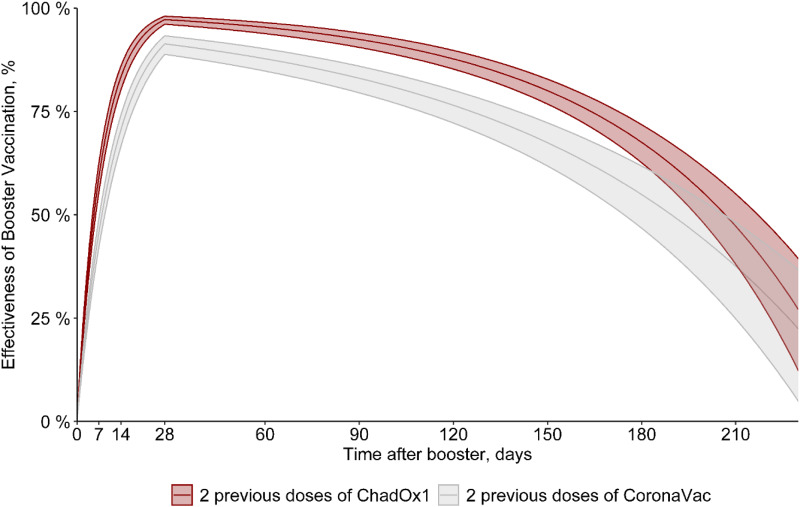
Effectiveness of 2 doses of Oxford-AstraZeneca (ChAdOx1) [red] or CoronaVac [gray] with a third (booster) dose with mRNA (Pfizer/BioNTech) vaccine, excluding those with previous COVID-19.

**Figure 2.**
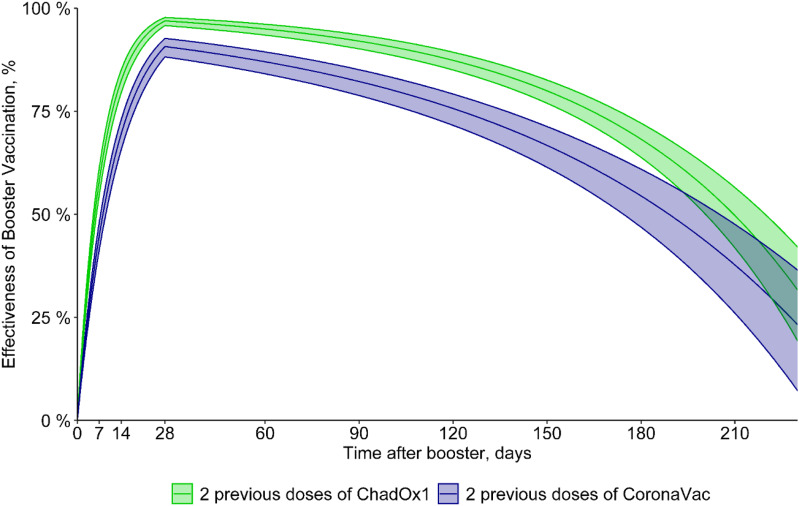
Effectiveness of 2 doses of Oxford-AstraZeneca (ChAdOx1) [green] or CoronaVac [purple] with a third (booster) dose with mRNA (Pfizer/BioNTech) vaccine, including those with previous COVID-19

Qualitative variables were characterized using absolute and relative frequencies in general and by interest groups. For comparisons, we used the χ^2^ or Fisher exact tests. Quantitative variables were described by medians, interquartile range (IQR, first and third quartiles), and minimum and maximum values due to the asymmetry observed in the variables,^15^ and comparisons were performed using nonparametric Mann-Whitney tests. Vaccine effectiveness was defined as 1 − hazard rate (HR),^16^ with HR determined by adjusting the survival analysis models with laboratory-confirmed COVID-19 as the outcome and vaccination and previous COVID-19 as the main explanatory variables. To assess the variation in the effectiveness of the booster dose over time (up to 250 days), we estimated the effectiveness rate by taking the log-risk ratio as a function of time (Figs. 1 and 2). These estimates considered sex, age, HCW job type (direct patient contact vs no direct patient contact), and comorbidities as covariates. HCWs with previous COVID-19 were excluded in the first model (Fig. 1), and they were included in the second model (Fig. 2). All analyses were performed using R software for statistical computing version 4.2.0 software,^17^ DOVE software,^18^ and ggplot2.^19^ All reported tests were 2-sided, and *P* < .01 was considered significant. The study was approved by the Hospital Israelita Albert Einstein Ethics Committee (CAAE 47110421.7.0000.0071), and the need for informed consent was waived.

## Results

During the study period, 18,359 individuals were screened for eligibility and 14,532 HCWs met inclusion criteria (Supplementary Appendix 1). Most were female (70.7%), and the median age was 36 years. Of those included, 892 (6.1%) received 2 doses of CoronaVac vaccine, 6,285 (43.3%) received 2 doses of CoronaVac vaccine plus mRNA (Pfizer/BioNTech) booster, 1,111 (7.6%) received 2 doses of ChAdOx1 vaccine, and 6,244 (43.0%) received 2 doses of ChAdOx1 vaccine plus mRNA (Pfizer/BioNTech) booster.

Compared to the group that received 2 doses of CoronaVac vaccine, the group that received a Pfizer/BioNTech booster following CoronaVac vaccine was significantly older and had a greater proportion of HCWs with patient contact (Table 1). Compared to the group that received 2 doses of ChAdOx1 vaccine, the group that received a Pfizer/BioNTech booster following a ChAdOx1 primary series was significantly older, had a greater proportion of women, and had a smaller proportion of HCWs with patient contact.

**Table 1.** Baseline Characteristics of Study Participants

Characteristic	Total	2 Doses of CoronaVac	2 Doses of CoronaVac and 1 Pfizer	*P* Value, 2 vs 3 CoronaVac	2 Doses of ChAdOx1	2 Doses of ChAdOx1 and 1 Pfizer	P Value, 2 vs 3 ChAdOx1
No. of participants	14,532	892	6,285		1,111	6,244	
Sex, no. (%)				.648			.004
Female	10,277 (70.7)	651 (73.0)	4,537 (72.2)		727 (65.4)	4,362 (69.9)	
Male	4,255 (29.3)	241 (27.0)	1,748 (27.8)		384 (34.6)	1,882 (30.1)	
Age, median				<.001			<.001
Median (IQR)	36 [29–42]	35 [28–40]	36 [30–42]		33 [25–40]	36 [29–42]	
Minimum - Maximum	18–83	18–69	18–82		18–75	18–83	
Missing	5	0	3		0	2	
Age ≥61 y, no. (%)	152 (1.0)	5 (0.6)	70 (1.1)	.178	15 (1.4)	62 (1.0)	.359
Job type, no. (%)				<.001			.003
No direct patient contact	7,272 (50.0)	267 (29.9)	1,461 (23.2)		797 (71.7)	4,747 (76.0)	
Direct patient facing	7,260 (50.0)	625 (70.1)	4,824 (76.8)		314 (28.3)	1,497 (24.0)	
COVID-19 (by PCR) prior to vaccination, no. (%)	2,662 (18.3)	123 (13.8)	1,198 (19.1)	<.001	168 (15.1)	1,173 (18.8)	.004
COVID-19 (by PCR), no. (%)	3,785 (26.0)	502 (56.3)	1,455 (23.2)	<.001	412 (37.1)	1,416 (22.7)	<.001
Follow-up period, days							
Median (IQR)	243 [193–273]	222 [131–329]	254 [216–289]	.046	248 [166–346]	229 [191–256]	<.001
Minimum–maximum	15–534	15–534	15–356		15–482	16–336	
Hospitalization, no. (%)	14 (0.10)	7 (0.80)	2 (0.03)	<.001^a^	2 (0.18)	3 (0.05)	.167^a^
ICU, no. (%)	2 (0.01)	2 (0.23)	0 (0.0)	.015^a^	0 (0.0)	0 (0.0)	…
Mechanical ventilation, no. (%)	1 (0.01)	1 (0.11)	0 (0.0)	.124^a^	0 (0.0)	0 (0.0)	…
Length of hospital stay, days				.184			
Median [IQR]	6 [3–10]	10 [5–11]	3 [3–4]		6 [5–6]	4 [4–5]	.374
Minimum–maximum	2–15	2–15	2–4		5–6	3–6	
No.	14	7	2		2	3	
Comorbidity, no. (%)^b^	3,726 (26.7)	193 (22.9)	1,558 (25.9)	.068	245 (24.5)	1,730 (28.3)	.016
Obesity, no. (%)^b^	1,288 (9.2)	70 (8.3)	512 (8.5)	.894	81 (8.1)	625 (10.2)	.045
Hypertension, no. (%)^b^	1,143 (8.2)	55 (6.5)	458 (7.6)	.292	69 (6.9)	561 (9.2)	.023
Dyslipidemia, no. (%)^b^	905 (6.5)	46 (5.5)	413 (6.9)	.145	48 (4.8)	398 (6.5)	.048
Asthma, no. (%)^b^	583 (4.2)	38 (4.5)	242 (4.0)	.565	36 (3.6)	267 (4.4)	.310
Diabetes mellitus, no. (%)^b^	341 (2.4)	17 (2.0)	139 (2.3)	.681	21 (2.1)	164 (2.7)	.339
Cancer, no. (%)^b^	311 (2.2)	14 (1.7)	137 (2.3)	.310	14 (1.4)	146 (2.4)	.067
Bronchitis, emphysema, or COPD, no. (%)^b^	277 (2.0)	14 (1.7)	74 (1.2)	.380	35 (3.5)	154 (2.5)	.090
Arthritis, no. (%)^b^	149 (1.1)	3 (0.4)	62 (1.0)	.089	12 (1.2)	72 (1.2)	>.99
Stroke, no. (%)^b^	34 (0.2)	5 (0.6)	10 (0.2)	.036	0 (0.0)	19 (0.3)	.097^a^
Chronic kidney disease, no. (%)^b^	19 (0.1)	1 (0.1)	2 (0.0)	.325^a^	3 (0.3)	13 (0.2)	.483^a^

Note. ChAdOx1 vaccine, Oxford-AstraZeneca vaccine; COPD, chronic obstructive pulmonary disease; COVID-19, coronavirus disease 2019; ICU, intensive care unit; IQR: interquartile range; PCR, polymerase chain reaction.

a Comparisons for categorical variables performed with χ^2^ or Fisher exact test; for quantitative variables, the Mann-Whitney test was used.

b Information available for 13,975 participants (96.2%), 843 with 2 doses of CoronaVac vaccine, 998 with 2 doses of ChAdOx1 vaccine, 6,018 with 2 doses of CoronaVac vaccine + Pfizer/BioNTech vaccine and 6,116 with 2 doses of ChAdOx1 vaccine + Pfizer/BioNTech vaccine.

During the study period, 2,662 were diagnosed with COVID-19 prior to vaccination, and 3,785 had COVID-19 after vaccination. Overall, COVID-19 cases detected after vaccination occurred in 56.3% of HCWs who received 2 doses of the CoronaVac vaccine and 23.2% who received a booster (*P* < .001). COVID-19 cases detected after vaccination occurred in 37.1% of HCWs who received 2 doses of the ChAdOx1 vaccine and 22.7% who received a booster (*P* < .001).

Estimates of vaccine effectiveness of 2 doses of Oxford-AstraZeneca (ChAdOx1) or CoronaVac with a third (booster) dose with mRNA (Pfizer/BioNTech) vaccine are shown in Figure 1 and Table 2. The estimated vaccine effectiveness rates in the period beginning 15 days after receiving the mRNA booster dose were 73% for HCWs who received primary doses of the CoronaVac vaccine and 85% those for who received primary doses of the ChAdOx1 vaccine. The highest vaccine effectiveness rates (peak level) were observed 30 days after the booster dose (91% and 97%, respectively), with a progressive decrease in vaccine effectiveness after this period. At 180 days after a booster, the vaccine effectiveness rates were 55% and 67.5%, respectively (Fig. 1 and Table 2). In addition, 0.8% of HCWs who received 2 doses of CoronaVac had at least 1 hospitalization compared to 0.03% of HCWs who received a booster dose following CoronaVac vaccine (*P* < .001). On the other hand, no difference was observed in hospitalizations between those with 2 doses of the ChAdOx1 vaccine (2 cases, 0.18%) and those with a booster dose following ChAdOx1 vaccine (3 cases, 0.05%) (*P* = .16). There was no statistically significant difference between those with 2 doses (either ChAdOx1 or CoronaVac) and a booster dose in length of stay, ICU stays, or mechanical ventilation use (Table 1). Only 1 HCW vaccinated with 2 doses of the ChAdOx1 vaccine died during the study period, before the booster dose was released. This HCW was immunocompromised due to systemic lupus erythematosus treatment. When including those with COVID-19 prior to vaccinationin in the model, the vaccine effectiveness was quite similar to the first vaccine effectiveness model in which those with prior infection were excluded. The estimated vaccine effectiveness in those receiving the mRNA booster dose was 72% for HCWs who received primary doses of the CoronaVac vaccine and 84.5% those for who received primary doses of the ChAdOx1 vaccine. The highest vaccine effectiveness (peak level) was observed after 30 days (90.5% and 97%, respectively) with a progressive decrease in vaccine effectiveness after this period, and at 180 days, the vaccine effectiveness was 54.5% and 68%, respectively (Fig. 2 and Table 3).

**Table 2.** Estimated Effectiveness of a Third COVID-19 Vaccine Dose (Pfizer/BioNTech vaccine) in Reducing the Risk of Infection Over Time Among Healthcare Workers (HCWs) Without Previous COVID-19

Days After Third Dose	2 Previous Doses of the CoronaVac Vaccine	2 Previous Doses of the ChAdOx1 Vaccine	Days After Third Dose	2 Previous Doses of the CoronaVac Vaccine	2 Previous Doses of the ChAdOx1 Vaccine
	VE % (95% CI)	VE % (95% CI)		VE % (95% CI)	VE % (95% CI)
0	0.0 (0.0–0.0)	0.0 (0.0–0.0)	130	73.8 (69.1–77.7)	85.5 (82.9–87.8)
5	35.4 (32.4–38.3)	47.3 (44.0–50.4)	135	72.3 (67.5–76.5)	84.3 (81.5–86.7)
10	58.3 (54.3–61.9)	72.2 (68.6–75.4)	140	70.8 (65.7–75.1)	83.0 (80.1–85.5)
15	73.0 (69.1–76.5)	85.4 (82.4–87.8)	145	69.2 (63.8–73.7)	81.6 (78.5–84.2)
20	82.6 (79.1–85.5)	92.3 (90.1–94.0)	150	67.4 (61.8–72.2)	80.0 (76.8–82.8)
25	88.7 (85.9–91.0)	95.9 (94.5–97.0)	155	65.6 (59.7–70.7)	78.3 (74.9–81.3)
30	91.2 (88.6–93.1)	97.1 (96.0–98.0)	160	63.7 (57.4–69.1)	76.5 (72.9–79.7)
35	90.7 (88.0–92.7)	96.9 (95.7–97.8)	165	61.7 (55.0–67.4)	74.5 (70.6–77.9)
40	90.1 (87.4–92.3)	96.6 (95.4–97.6)	170	59.5 (52.4–65.6)	72.4 (68.2–76.0)
45	89.6 (86.8–91.8)	96.4 (95.0–97.3)	175	57.3 (49.7–63.7)	70.1 (65.5–74.0)
50	89.0 (86.2–91.3)	96.0 (94.7–97.1)	180	54.9 (46.8–61.8)	67.5 (62.6–71.8)
55	88.4 (85.5–90.7)	95.7 (94.3–96.8)	185	52.4 (43.7–59.8)	64.8 (59.4–69.5)
60	87.7 (84.8–90.1)	95.3 (93.8–96.5)	190	49.7 (40.4–57.6)	61.8 (55.9–67.0)
65	87.1 (84.0–89.5)	95.0 (93.4–96.2)	195	46.9 (36.8–55.4)	58.6 (52.0–64.3)
70	86.3 (83.2–88.9)	94.5 (92.9–95.8)	200	44.0 (33.1–53.1)	55.1 (47.8–61.4)
75	85.6 (82.3–88.2)	94.1 (92.3–95.4)	205	40.8 (29.1–50.7)	51.3 (43.1–58.3)
80	84.8 (81.5–87.5)	93.6 (91.8–95.0)	210	37.5 (24.8–48.1)	47.2 (38.1–55.1)
85	83.9 (80.5–86.7)	93.0 (91.2–94.5)	215	34.1 (20.3–45.4)	42.8 (32.5–51.5)
90	83.0 (79.5–85.9)	92.4 (90.5–94.0)	220	30.4 (15.5–42.7)	38.0 (26.3–47.8)
95	82.1 (78.5–85.1)	91.8 (89.8–93.4)	225	26.5 (10.4–39.7)	32.7 (19.6–43.7)
100	81.1 (77.3–84.2)	91.1 (89.0–92.8)	230	22.4 (4.9–36.7)	27.1 (12.3–39.4)
105	80.0 (76.2–83.3)	90.4 (88.2–92.1)	235	18.1 (0–33.5)	20.9 (4.2–34.7)
110	78.9 (74.9–82.3)	89.5 (87.3–91.4)	240	13.5 (0–30.2)	14.2 (0–29.7)
115	77.7 (73.6–81.2)	88.7 (86.3–90.6)	245	8.7 (0–26.7)	7.0 (0–24.4)
120	76.5 (72.2–80.1)	87.7 (85.3–89.8)	250	3.6 (0–23.0)	0 (0–18.7)
125	75.2 (70.7–79.0)	86.7 (84.1–88.8)			

Note. These estimates considered sex, age, HCW job, and comorbidities as covariates. Cases with previous COVID-19 were excluded.

**Table 3.** Estimated Effectiveness of a Third COVID-19 Vaccine Dose (Pfizer/BioNTech vaccine) in Reducing the Risk of Infection Over Time Among Healthcare Workers (HCWs) Including Those With Previous COVID-19

Days after Third Dose	2 Previous Doses of the CoronaVac Vaccine	2 Previous Doses of the ChAdOx1 Vaccine	Days After Third Dose	2 Previous Doses of the CoronaVac Vaccine	2 Previous Doses of the ChAdOx1 Vaccine
	VE % (95% CI)	VE % (95% CI)		VE (95% CI)	VE % (95% CI)
0	0.0 (0.0–0.0)	0.0 (0.0–0.0)	130	73.0 (68.6–76.8)	85.2 (82.7–87.4)
5	34.6 (31.7–37.3)	46.3 (43.2–49.2)	135	71.5 (66.9–75.5)	84.1 (81.4–86.3)
10	57.2 (53.4–60.7)	71.1 (67.7–74.2)	140	70.0 (65.2–74.2)	82.8 (80.1–85.2)
15	72.0 (68.2–75.3)	84.5 (81.6–86.9)	145	68.4 (63.4–72.8)	81.4 (78.6–83.9)
20	81.7 (78.3–84.5)	91.7 (89.6–93.3)	150	66.7 (61.4–71.3)	79.9 (76.9–82.6)
25	88.0 (85.1–90.3)	95.5 (94.1–96.6)	155	64.9 (59.3–69.8)	78.3 (75.2–81.1)
30	90.5 (88.0–92.5)	96.8 (95.7–97.7)	160	63.0 (57.1–68.1)	76.6 (73.3–79.6)
35	90.0 (87.4–92.0)	96.6 (95.4–97.5)	165	61.1 (54.8–66.5)	74.8 (71.2–77.9)
40	89.4 (86.8–91.6)	96.3 (95.0–97.2)	170	59.0 (52.3–64.7)	72.8 (68.9–76.1)
45	88.9 (86.2–91.1)	96.0 (94.7–97.0)	175	56.8 (49.7–62.9)	70.6 (66.5–74.2)
50	88.3 (85.5–90.5)	95.7 (94.3–96.7)	180	54.5 (46.9–61.0)	68.2 (63.8–72.1)
55	87.7 (84.8–90.0)	95.3 (93.9–96.4)	185	52.0 (43.9–59.0)	65.7 (60.9–69.9)
60	87.0 (84.1–89.4)	95.0 (93.5–96.1)	190	49.5 (40.8–56.9)	63.0 (57.7–67.6)
65	86.3 (83.3–88.8)	94.6 (93.0–95.8)	195	46.7 (37.4–54.7)	60.0 (54.3–65.1)
70	85.6 (82.5–88.1)	94.1 (92.5–95.4)	200	43.9 (33.8–52.4)	56.8 (50.5–62.4)
75	84.8 (81.6–87.4)	93.6 (92.0–95.0)	205	40.9 (30.0–50.0)	53.4 (46.3–59.5)
80	84.0 (80.8–86.7)	93.1 (91.4–94.5)	210	37.7 (26.0–47.6)	49.7 (41.8–56.5)
85	83.1 (79.8–85.9)	92.6 (90.8–94.0)	215	34.4 (21.7–45.0)	45.7 (36.9–53.2)
90	82.2 (78.8–85.1)	92.0 (90.1–93.5)	220	30.9 (17.2–42.3)	41.4 (31.6–49.8)
95	81.3 (77.8–84.2)	91.4 (89.4–93.0)	225	27.1 (12.4–39.4)	36.7 (25.7–46.0)
100	80.3 (76.7–83.3)	90.7 (88.7–92.3)	230	23.2 (7.2–36.5)	31.6 (19.3–42.1)
105	79.2 (75.5–82.3)	89.9 (87.8–91.7)	235	19.1 (1.8–33.4)	26.2 (12.4–37.8)
110	78.1 (74.3–81.3)	89.1 (87.0–90.9)	240	14.8 (0–30.2)	20.3 (4.8–33.3)
115	76.9 (73.0–80.3)	88.3 (86.0–90.2)	245	10.2 (0–26.8)	14.0 (0–28.5)
120	75.7 (71.6–79.2)	87.3 (85.0–89.3)	250	5.4 (0–23.3)	7.1 (0–23.3)
125	74.4 (70.1–78.0)	86.3 (83.9–88.4)			

Note. These estimates considered sex, age, HCW job, comorbidities and previous COVID-19 as covariates.

### Whole-genome sequencing analysis

During the study period, 430 SARS-CoV-2 samples, the first collected from each HCW, were screened for mutations. One (0.2%) case was the SARS-CoV-2 alpha variant, 68 (15.8%) were P1 strain (Gamma SARS-CoV-2 variant), 213 (49.5%) were delta variant cases, and 147 (34.2%) were omicron variant cases. In November and December 2021, 90.5% of cases were the delta variant. Almost all cases (95.2%) were the omicron variant in January and February 2022, and 100% were the omicron variant from March to July 2022 (Table 4).

**Table 4.** Participants with SARS-CoV-2 Variants of Concern (n=430) Detected by Whole-Genome Sequencing

Period	SARS-CoV-2 VOC Variant Lineage, No. (%)^a^					WGS Cases, No.
	Alpha	B.1.1.28	Delta	Gamma	Omicron	
Mar–Apr 2021	1 (5.0)		1 (5.0)	18 (90.0)		20
May–Jun 2021		1 (50.0)		1 (50.0)		2
Jul–Aug 2021			47 (49.0)	49 (51.0)		96
Sep–Oct 2021			139 (100.0)			139
Nov–Dec 2021			19 (90.5)		2 (9.5)	21
Jan–Feb 2022			7 (4.9)		137 (95.1)	144
Mar–Apr2022					2 (100.0)	2
May–Jun 2022					5 (100.0)	5
Jul 2022					1 (100.0)	1
Total	1 (0.2)	1 (0.2)	213 (49.5)	68 (15.8)	147 (34.2)	430

Note. VOC, variant of concern; WGS, whole-genome sequencing.

a Data presented as number (row percentage). The 430 samples screened for mutations were the first samples collected from each individual.

## Discussion

In this retrospective study, longer-term vaccine effectiveness with heterologous COVID-19 vaccines (2 doses of either CoronaVac or ChAdOx1 vaccine followed by mRNA booster) was still reasonable for both vaccines, even after adjusting for important variables such as the time to event from the last dose (ie, exposure duration) and infection with the SARS-CoV-2 delta and omicron variants. However, protection after 3 doses of the COVID-19 vaccine waned overtime but provided good protection against infection, hospitalization and death, especially up to 6 months.

This result is endorsed by published evidence that the booster is capable of raising the protection already built by the primary doses. Rates of COVID-19 were significantly lower among people who received the booster than among those who have just been vaccinated with 2 doses.^13,20,21^ A similar comparison of 3 versus 2 doses of SARS-CoV-2 vaccines found a high vaccine effectiveness of booster dose against symptomatic infection and demonstrated that the booster was safe.^22^ A case–control study corroborates that the booster adds significant protection.^23^ However, this study showed reduced vaccine effectiveness during the SARS-CoV-2 omicron variant period.^23^ Notably, these booster studies did not consider administration of heterologous COVID-19 vaccines in their analysis.

This waning immunogenicity of the vaccines over time was also reported in another study that evaluated the duration and effectiveness of immunity induced by 2 doses of mRNA vaccine and 2 doses of viral vector vaccine.^24^ In the short term, the 2 doses promoted high protection against SARS-CoV-2 infection (vaccine effectiveness, 91%–97%). However, after 6 months, the protection decreased (vaccine effectiveness, 55%–67%) and in this period of waning of protection, the omicron variant was the predominant SARS-CoV-2 strain. This finding reinforces the importance of the booster doses after the primary doses of the vaccines to maintain protection, considering not only the waning immunogenicity but also the emergence of the new variants. Initial studies have shown that heterologous boostering may result in higher neutralizing-antibody responses than homologous boostering, particularly after primary doses with a viral vector vaccine.^25,26^ In a study of US veterans, heterologous mRNA boosting offered better protection against COVID-19 in individuals who were initially vaccinated with a viral vector vaccine.^27^ A Chilean study also demonstrated heterologous boosters showed higher vaccine effectiveness among individuals with a complete primary vaccination doses with CoronaVac for all outcomes, providing additional support for this vaccine strategy.^28^ Similarly, a recent study from Malaysa demonstrated that heterologous boosting using Pfizer/BioNTech vaccine after inactivated and viral vector primary vaccination is preferred.^29^ Thus, HCWs who received either Coronavac or ChAdOx1 primary vaccination should receive an mRNA booster, if available.

In our study, vaccine effectiveness among HCWs who had previous COVID-19 was similar to vaccine effectiveness among HCWs who did not have previous infection, and vaccine effectiveness among both groups declined over time. Therefore, the second booster should be recommended to HCWs who completed 3 doses regardless of whether they had COVID-19. In addition, vaccine effectiveness was nearly zero by the ninth month after the first booster, and a second booster is strongly encouraged for HCWs before the protection from prior vaccines completely fades away.

Our study had several limitations. First, this is an observational study, which is subject to multiple biases.^30^ However, this is the most common study design in the infection prevention literature.^30^ We did not perform a test-negative design case–control study because this study was retrospectively conducted using data from symptom-based testing. There is a possibility that HCWs had asymptomatic SARS-CoV-2 infection and did not undergo testing, leading to misclassification of the outcome.^31,32^ Second, we did not directly compare vaccine effectiveness between those with 2 doses of CoronaVac followed by mRNA vaccine and those with 2 doses of ChAdOx1 followed by mRNA vaccine. However, estimated vaccine effectiveness over time was comparable between the 2 groups with >50% of vaccine effectiveness up to 180 days after receiving the mRNA booster dose, thus a good time point to get a second booster (Figs. 1 and 2). Third, we were not able to compare homologous versus heterologous booster because most of our HCWs received heterologous COVID-19 vaccination. A previous study evaluating longer-term vaccine effectiveness detected that heterologous boosting was associated with greater protection than homologous boosting for those with mRNA vaccine primary dosing.^33^ However, a nationwide study from Brazil detected reduced longer-term vaccine effectiveness for homologous and heterologous (Pfizer/BioNTech COVID-19 vaccine) booster doses in preventing COVID-19 in adults who received primary doses of CoronaVac during the SARS-CoV-2 omicron variant period.^34^ Fourth, we could not perform further analyses by immunocompromised status due to the limited number of cases. Fifth, neutralizing viral antigen-binding antibody levels were not available in our HCW cohort study. However, the US FDA does not recommend antibody testing for SARS-CoV-2 to determine immunity or protection from COVID-19, especially among those who are vaccinated.^35^ Sixth, our study focused only on long-term vaccine effectiveness for the third dose against COVID-19 in HCWs; thus, we could not fully evaluate vaccine effectiveness for other outcomes such as COVID-19 hospitalization, COVID-19 reinfection, or COVID-19 death, because these outcomes were few in number. Other studies have demonstrated that the booster dose also has a significant protective effect against these severe outcomes.^11,36^ Lastly, we were not able to perform viral sequencing in all COVID-19 cases; it was limited to a small random sample of 430 HCWs, but the most prevalent variants detected in the study period were SARS-CoV-2 delta and omicron variants, representing ∼85% of cases.

In conclusion, viral vector and inactivated virus COVID-19 vaccines can significantly prevent infection, hospitalization, and death among HCWs when boosted with a third dose of Pfizer/BioNTech mRNA vaccine, even for a relatively long period (6 months). This heterologous vaccine strategy was also effective among HCWs even after emergence of a new SARS-CoV-2 variants (ie, omicron). The associated protection waned over 180 days, independent of having previous COVID-19, which suggests the necessity for a second booster. More studies are needed to evaluate vaccine effectiveness for other heterologous prime-booster COVID-19 vaccines (bivalent COVID-19 vaccines), COVID-19 breakthrough infection, and analysis of genomic surveillance to better understand vaccine effectiveness against newer SARS-CoV-2 variants, such as omicron BA.5 and XBB.1.5.

## Supplementary material

For supplementary material accompanying this paper visit http://doi.org/10.1017/ash.2023.173. click here to view supplementary material

## References

[1] Brown CM , Vostok J , Johnson H , et al. Outbreak of SARS-CoV-2 infections, including COVID-19 vaccine breakthrough infections, associated with large public gatherings—Barnstable County, Massachusetts, July 2021. Morbid Mortal Wkly Rep 2021;70:1059–1062. 10.15585/mmwr.mm7031e2 PMC8367314 34351882

[2] Glatman-Freedman A , Hershkovitz Y , Kaufman Z , Dichtiar R , Keinan-Boker L , Bromberg M. Effectiveness of BNT162b2 vaccine in adolescents during outbreak of SARS-CoV-2 delta variant infection, Israel, 2021. Emerg Infect Dis 2021;27:2919–2922. 34570694 10.3201/eid2711.211886 PMC8544958

[3] Lopez Bernal J , Andrews N , Gower C , et al. Effectiveness of the Pfizer-BioNTech and Oxford-AstraZeneca vaccines on covid-19 related symptoms, hospital admissions, and mortality in older adults in England: test negative case–control study. BMJ Clin Res 2021;373:n1088. 10.1136/bmj.n1088 PMC8116636 33985964

[4] Haas EJ , Angulo FJ , McLaughlin JM , et al. Impact and effectiveness of mRNA BNT162b2 vaccine against SARS-CoV-2 infections and COVID-19 cases, hospitalisations, and deaths following a nationwide vaccination campaign in Israel: an observational study using national surveillance data. Lancet 2021;397:1819–1829. 33964222 10.1016/S0140-6736(21)00947-8 PMC8099315

[5] National Center for, Immunization Respiratory Diseases. Division of Viral, Diseases. Interim public health recommendations for fully vaccinated people. Centers for Disease Control and Prevention website. https://stacks.cdc.gov/view/cdc/108355. Published 2021. Accessed April 4, 2022.

[6] Smeltzer SC , Copel LC , Bradley PK , et al. Vulnerability, loss, and coping experiences of health care workers and first responders during the COVID-19 pandemic: a qualitative study. Int J Qual Stud Health Well-Being 2022;17:2066254. 35442177 10.1080/17482631.2022.2066254 PMC9037221

[7] Galanis P , Vraka I , Fragkou D , Bilali A , Kaitelidou D. Impact of personal protective equipment use on health care workers’ physical health during the COVID-19 pandemic: a systematic review and meta-analysis. Am J Infect Control 2021;49:1305–1315. 33965463 10.1016/j.ajic.2021.04.084 PMC8102386

[8] Lee T , Kwon HD , Lee J. The effect of control measures on COVID-19 transmission in South Korea. PloS One 2021;16:e0249262. 33780504 10.1371/journal.pone.0249262 PMC8006988

[9] Majra D , Benson J , Pitts J , Stebbing J. SARS-CoV-2 (COVID-19) superspreader events. J Infect 2021;82:36–40. 33245943 10.1016/j.jinf.2020.11.021 PMC7685932

[10] Marra AR , Miraglia JL , Malheiros DT , et al. Effectiveness of two COVID-19 vaccines (viral vector and inactivated viral vaccine) against SARS-CoV-2 infection in a cohort of healthcare workers. Infect Control Hosp Epidemiol 2023;44:75–81. 35351217 10.1017/ice.2022.50 PMC9002147

[11] Arbel R , Hammerman A , Sergienko R , et al. BNT162b2 vaccine booster and mortality due to COVID-19. N Engl J Med 2021;385:2413–2420. 34879190 10.1056/NEJMoa2115624 PMC8728797

[12] Barda N , Dagan N , Cohen C , et al. Effectiveness of a third dose of the BNT162b2 mRNA COVID-19 vaccine for preventing severe outcomes in Israel: an observational study. Lancet 2021;398:2093–2100. 34756184 10.1016/S0140-6736(21)02249-2 PMC8555967

[13] Marra AR , Miraglia JL , Malheiros DT , et al. Effectiveness of heterologous coronavirus disease 2019 (COVID-19) vaccine booster dosing in Brazilian healthcare workers, 2021. Clin Infect Dis 2023;76:e360–e366. 35639918 10.1093/cid/ciac430 PMC9213833

[14] Greninger AL , Naccache SN , Federman S , et al. Rapid metagenomic identification of viral pathogens in clinical samples by real-time nanopore sequencing analysis. Genome Med 2015;7:99. 26416663 10.1186/s13073-015-0220-9 PMC4587849

[15] Altman DG. Practical statistics for medical research. Boca Raton, FL: CRC Press; 1991.

[16] Nauta J. Statistics in clinical vaccine trials. New York: Springer Science & Business Media; 2010.

[17] R Core Team. R: A language and environment for statistical computing. 2021. http://www.R-project.org/. Accessed January 17, 2023.

[18] Lin DGY , Zeng D , Holloway ST . Package “DOVE”: durability of vaccine efficacy. 2022. R package version 1.9. https://cran.wustl.edu/web/packages/DOVE/DOVE.pdf. Published 2022. Accessed January 17, 2023.

[19] Wickham H. Ggplot2: Elegant Graphics for Data Analysis. Springer-Verlag New York. 2016.

[20] Munro APS , Janani L , Cornelius V , et al. Safety and immunogenicity of seven COVID-19 vaccines as a third dose (booster) following two doses of ChAdOx1 nCov-19 or BNT162b2 in the UK (COV-BOOST): a blinded, multicentre, randomised, controlled, phase 2 trial. Lancet 2021;398:2258–2276. 34863358 10.1016/S0140-6736(21)02717-3 PMC8639161

[21] Yigit M , Ozkaya-Parlakay A , Cosgun Y , Ince YE , Bulut YE , Senel E. Should a third booster dose be scheduled after two doses of CoronaVac? A single-center experience. J Med Virol 2022;94:287–290. 34487373 10.1002/jmv.27318 PMC8661641

[22] Butt AA , Talisa VB , Yan P , Shaikh OS , Omer SB , Mayr FB. Vaccine effectiveness of 3 versus 2 doses of severe acute respiratory syndrome coronavirus 2 (SARS-CoV-2) mRNA vaccines in a high-risk national population. Clin Infect Dis 2022;75:e579–e584. 35245940 10.1093/cid/ciac178 PMC8903438

[23] Richterman A , Behrman A , Brennan PJ , O’Donnell JA , Snider CK , Chaiyachati KH. Durability of severe acute respiratory syndrome coronavirus 2 messenger RNA booster vaccine protection against omicron among healthcare workers with a vaccine mandate. Clin Infect Dis 2023;76:e319–e326. 35666508 10.1093/cid/ciac454 PMC9214168

[24] Hall V , Foulkes S , Insalata F , et al. Protection against SARS-CoV-2 after COVID-19 vaccination and previous infection. N Engl J Med 2022;386:1207–1220. 35172051 10.1056/NEJMoa2118691 PMC8908850

[25] Normark J , Vikström L , Gwon YD , et al. Heterologous ChAdOx1 nCoV-19 and mRNA-1273 vaccination. N Engl J Med 2021;385:1049–1051. 34260850 10.1056/NEJMc2110716 PMC8314734

[26] Atmar RL , Lyke KE , Deming ME , et al. Homologous and heterologous COVID-19 booster vaccinations. N Engl J Med 2022;386:1046–1057. 35081293 10.1056/NEJMoa2116414 PMC8820244

[27] Mayr FB , Talisa VB , Shaikh O , Yende S , Butt AA. Effectiveness of homologous or heterologous COVID-19 boosters in veterans. N Engl J Med 2022;386:1375–1377. 35139265 10.1056/NEJMc2200415 PMC8849183

[28] Jara A , Undurraga EA , Zubizarreta JR , et al. Effectiveness of homologous and heterologous booster doses for an inactivated SARS-CoV-2 vaccine: a large-scale prospective cohort study. Lancet Global Health 2022;10:e798–e806. 35472300 10.1016/S2214-109X(22)00112-7 PMC9034854

[29] Suah JL , Tng BH , Tok PSK , et al. Real-world effectiveness of homologous and heterologous BNT162b2, CoronaVac, and AZD1222 booster vaccination against Delta and Omicron SARS-CoV-2 infection. Emerg Microbe Infect 2022;11:1343–1345. 10.1080/22221751.2022.2072773 PMC9132393 35499301

[30] Harris AD , Lautenbach E , Perencevich E. A systematic review of quasi-experimental study designs in the fields of infection control and antibiotic resistance. Clin Infect Dis 2005;41:77–82. 15937766 10.1086/430713

[31] Andrews N , Stowe J , Kirsebom F , et al. Effectiveness of COVID-19 booster vaccines against COVID-19-related symptoms, hospitalization and death in England. Nat Med 2022;28:831–837. 35045566 10.1038/s41591-022-01699-1 PMC9018410

[32] Cerqueira-Silva T , Katikireddi SV , de Araujo Oliveira V , et al. Vaccine effectiveness of heterologous CoronaVac plus BNT162b2 in Brazil. Nat Med 2022;28:838–843. 35140406 10.1038/s41591-022-01701-w PMC9018414

[33] Lin DY , Gu Y , Xu Y , et al. Association of primary and booster vaccination and prior infection with SARS-CoV-2 infection and severe COVID-19 outcomes. JAMA 2022;328:1415–1426. 36155617 10.1001/jama.2022.17876 PMC9513711

[34] Ranzani OT , Hitchings MDT , de Melo RL , et al. Effectiveness of an inactivated COVID-19 vaccine with homologous and heterologous boosters against omicron in Brazil. Nat Commun 2022;13:5536. 36202800 10.1038/s41467-022-33169-0 PMC9537178

[35] Antibody testing is not currently recommended to assess immunity after COVID-19 vaccination: FDA safety communication. Food and Drug Administration website. https://www.fda.gov/medical-devices/safety-communications/antibody-testing-not-currently-recommended-assess-immunity-after-covid-19-vaccination-fda-safety. Published 2021. Accessed May 12, 2022.

[36] Bar-On YM , Goldberg Y , Mandel M , et al. Protection of BNT162b2 vaccine booster against COVID-19 in Israel. N Engl J Med 2021;385:1393–1400. 34525275 10.1056/NEJMoa2114255 PMC8461568

