# Supplementary material for: Longer-term effectiveness of a heterologous coronavirus disease 2019 (COVID-19) vaccine booster in healthcare workers in Brazil
Source: Antimicrob Steward Healthc Epidemiol. 2023 Jun 22;3(1):e104. doi: 10.1017/ash.2023.173 (PMC10311693; doi:10.1017/ash.2023.173)
Supplement: Supplementary file 1 [file S2732494X23001730sup001.docx]

**Supplementary Appendix 1**. Reasons for HCWs not being eligible for the COVID-19 VE analysis

(3 doses vs. 2 doses)

| 18,359 HCWs | |  |
| --- | --- | --- |
|  |  |  |
|  |  | 2,508 HCWs were out of the hospital |
|  |  |  |
|  |  | 3 HCWs without any COVID-19 vaccine registration |
|  |  |  |
|  |  | 23 HCWs with one dose of COVID-19 vaccine |
|  |  |  |
|  |  | 144 HCWs with two doses of COVID-19 vaccine combinations out of the study scope |
|  |  |  |
|  |  | 463 HCWs with three doses of COVID-19 vaccine combinations out of the study scope** |
|  |  |  |
|  |  | 230 HCWs with one dose of COVID-19 vaccine in 2020 (Clinical Trial) |
|  |  |  |
|  |  | 72 HCWs contracted Covid-19 between 0 and 14 days of the COVID-19 vaccine’s first dose |
|  |  |  |
|  |  | 202 HCWs contracted COVID-19 infection between 15 days between the first and the second dose of the COVID-19 vaccine |
|  |  |  |
|  |  | 46 HCWs contracted COVID-19 between 0 and 14 days of COVID-19 vaccine second dose |
|  |  |  |
|  |  | 37 HCWs received the CoronaVac vaccine with less than 15 days between the COVID-19 vaccine doses (the first and the second dose) |
|  |  |  |
|  |  | 33 HCWs received ChAdOx1vaccine with less than 50 days between the COVID-19 vaccine doses (the first and the second dose) |
|  |  |  |
|  |  | 59 HCWs with less than 14 days of follow-up |
|  |  |  |
|  |  | 7 HCW with inconsistent data |
|  |  |  |
| 14,532 HCWs | | → 2^nd^ model analysis (with previous COVID-19 infection) |
|  |  |  |
|  |  | 2,662 with previous COVID-19 infection |
|  |  |  |
| 11,870 HCWs | | → 1^st^ model analysis (without previous COVID-19 infection) |

HCWs=Healthcare workers
